# Supplementary material for: Cross-sectional study of the ambulance transport between healthcare facilities with medical support via telemedicine: Easy, effective, and safe tool
Source: PLoS One. 2021 Sep 30;16(9):e0257801. doi: 10.1371/journal.pone.0257801 (PMC8483335; doi:10.1371/journal.pone.0257801)
Supplement: Attachment — Submitted filename: Response to reviewers.docx [file pone.0257801.s002.docx]

Response to reviewers

Reviewer’s comments:

1) Please include the dimensions of the display and show a couple of typical (de-identified) screen shots to illustrate the type of information being shared.

Answer: Display dimension and a screen shot of the de-identified data are included.

2) You refer to the checklist used by the nurses. This should be made available online. This is an important aspect of your paper and others will find this useful.

Answer: The checklist was included as figure 3

3) page 10: "the doctor received a boletin from the nurse ..." do you mean "Bulletin?"

Answer: The correction was made.

4) p. 11: "In this study, ambulance transport was considered safe ..." Consider replacing "safe" with "low risk." We are never completely safe.

Answer: Replacement done.

5) Please provide additional details regarding participant consent. In the Methods section, please ensure that you have specified (1) whether consent was informed and (2) what type you obtained (for instance, written or verbal). If your study included minors, state whether you obtained consent from parents or guardians. If the need for consent was waived by the ethics committee, please include this information.

Answer: The need for consent was waived by the ethics committee. We added this information in the Methods section.
